# Review on Driving Circuits for Wide-Bandgap Semiconductor Switching Devices for Mid- to High-Power Applications

**DOI:** 10.3390/mi12010065

**Published:** 2021-01-08

**Authors:** Chao-Tsung Ma, Zhen-Huang Gu

**Affiliations:** Department of Electrical Engineering, CEECS, National United University, Miaoli 36063, Taiwan; M0621002@smail.nuu.edu.tw

**Keywords:** wide-bandgap (WBG), gallium nitride (GaN), silicon carbide (SiC), high electron mobility transistor (HEMT), metal-oxide-semiconductor field effect transistor (MOSFET), driving technology

## Abstract

Wide-bandgap (WBG) material-based switching devices such as gallium nitride (GaN) high electron mobility transistors (HEMTs) and silicon carbide (SiC) metal-oxide-semiconductor field-effect transistors (MOSFETs) are considered very promising candidates for replacing conventional silicon (Si) MOSFETs for various advanced power conversion applications, mainly because of their capabilities of higher switching frequencies with less switching and conduction losses. However, to make the most of their advantages, it is crucial to understand the intrinsic differences between WBG- and Si-based switching devices and investigate effective means to safely, efficiently, and reliably utilize the WBG devices. This paper aims to provide engineers in the power engineering field a comprehensive understanding of WBG switching devices’ driving requirements, especially for mid- to high-power applications. First, the characteristics and operating principles of WBG switching devices and their commercial products within specific voltage ranges are explored. Next, considerations regarding the design of driving circuits for WBG switching devices are addressed, and commercial drivers designed for WBG switching devices are explored. Lastly, a review on typical papers concerning driving technologies for WBG switching devices in mid- to high-power applications is presented.

## 1. Introduction

**Figure 1:**
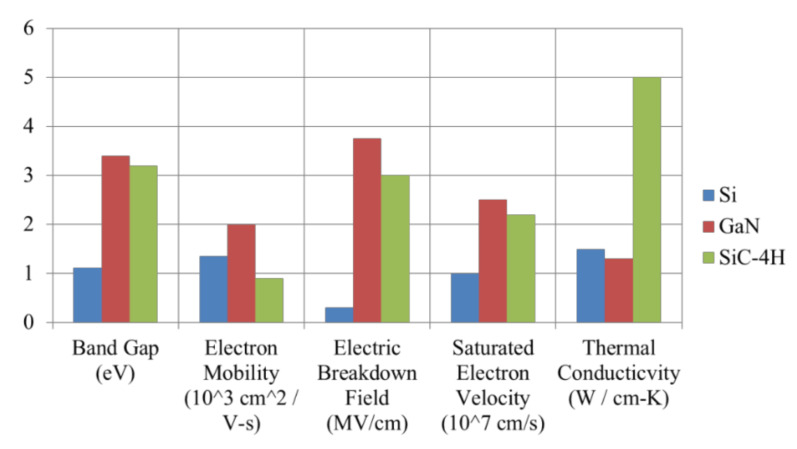
Comparison of Si, gallium nitride (GaN), and silicon carbide (SiC).

In modern industries, requirements for the performance of various power electronic-based converters are becoming stricter in terms of capacity, voltage level, efficiency, and size (switching frequency related issues). In order to enhance the performance of existing power converters, replacing conventional Si switching devices with wide-bandgap (WBG) switching devices such as gallium nitride (GaN) high electron mobility transistors (HEMTs) and silicon carbide (SiC) metal-oxide-semiconductor field-effect transistors (MOSFETs) is currently a popularly adopted method. WBG semiconductor materials offer superior characteristics to those of Si, as shown in Figure 1. The respective merits of GaN and SiC lead to the advantageous adoption of GaN HEMTs for low (<1 kW) to mid (<10 kW) power applications and SiC MOSFETs for mid (<10 kW) to high (>10 kW) power applications in practical design scenarios. The superiority of GaN HEMTs is yet to be fully utilized because they feature some form of heterogeneous integration with dissimilar substrate. This leads to large thermal boundary resistance between GaN and substrate, causing the self-heat issue [1], which may cause the switching device to overheat. However, GaN HEMTs offer the highest efficiency and switching speed, and SiC MOSFETs provide the highest voltage, current, and temperature capabilities. The main challenge of using the WBG semiconductor switching devices is overcoming potential difficulties introduced from their high slew rates, which could worsen electromagnetic interference (EMI) level and may cause voltage oscillation and instability [2,3,4,5,6,7].

**Figure 2:**
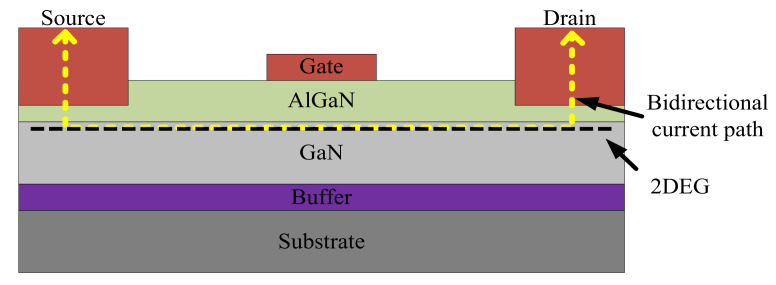
D-mode GaN high electron mobility transistors (HEMT).

**Figure 3:**
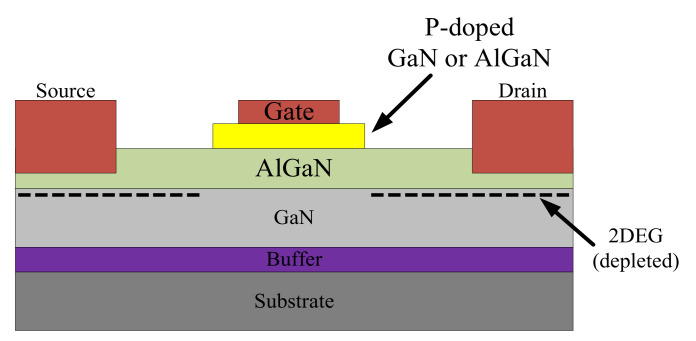
E-mode GaN HEMT.

**Figure 4:**
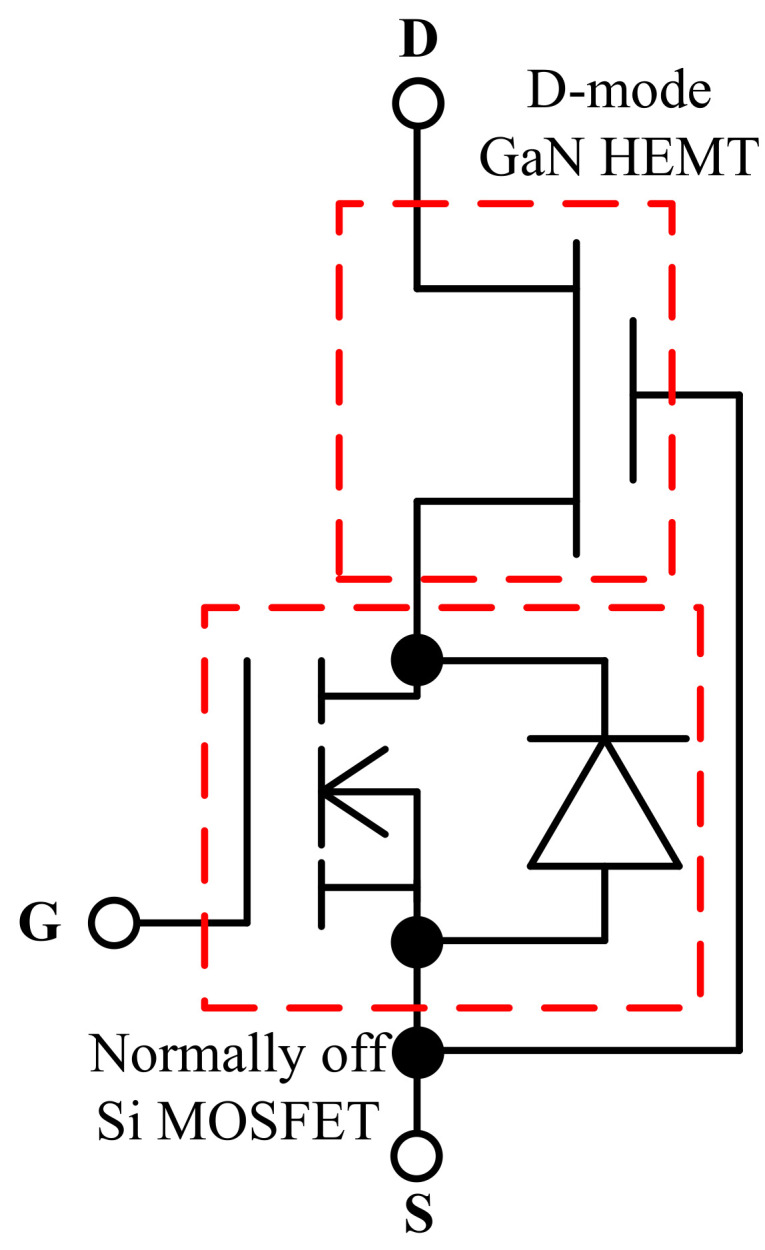
Cascode GaN HEMT.

The GaN HEMT is designed with a unique aluminum gallium nitride (AlGaN)/GaN heterojunction structure where two-dimensional electron gas (2DEG) is formed. The 2DEG allows large bidirectional current and yields extremely low on resistance. GaN HEMTs are currently divided into three types: depletion mode (D-mode), enhancement mode (E-mode), and cascode devices. The D-mode GaN HEMT, as shown in Figure 2, is naturally on because of the 2DEG and can be turned off with negative gate-source voltage. The E-mode GaN HEMT, as shown in Figure 3, is normally off because the 2DEG has been depleted by an additional P-doped layer of GaN or AlGaN on the gate, and it can be turned on with appropriate gate-source voltage. The cascode GaN HEMT, as shown in Figure 4, is also normally off because it consists of a D-mode GaN HEMT and an additional high-speed low-voltage Si MOSFET, and it can be turned on with appropriate gate-source voltage applied on the Si MOSFET. E-mode and cascode GaN HEMTs possess different characteristics mainly because of the additional Si MOSFET in the cascode device: the E-mode device offers lower on resistance, higher operating temperature, and no body diode, while the cascode device offers less strict driving requirements, as shown in Table 1 [4,5,6,7].

**Figure 5:**
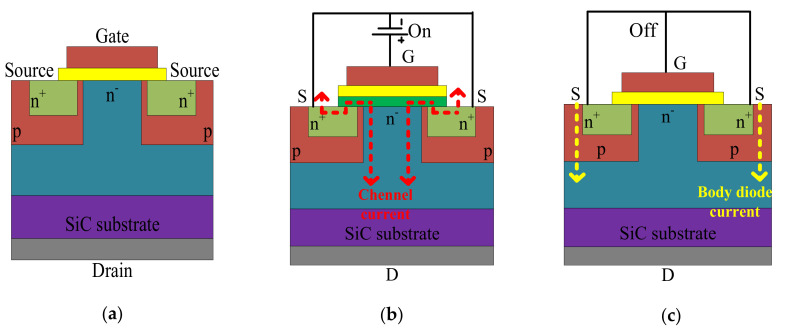
Schematic diagrams of a SiC MOSFET, (**a**) basic structure, (**b**) the gate-source voltage and current path in on-state, (**c**) the gate-source voltage and current path in off-state.

The SiC MOSFET has a similar structure to that of Si MOSFET, as shown in Figure 5, but the thickness can be made an order smaller because of SiC’s higher voltage capability. This leads to much smaller on resistance (although not as small as that of the GaN HEMT). Additionally, the SiC MOSFET offers the highest power capability. The operation of the SiC MOSFET is the same as that of the Si MOSFET: with appropriate gate-source voltage, the device can be turned on, and the body diode is used for reverse conduction during off state [8,9]. A general comparison of Si MOSFET, normally off GaN HEMTs, and SiC MOSFET is shown in Table 2.

In recent years, because of the high slew rate of the WBG semiconductor switching devices, the philosophy of replacing conventional Si devices with WBG devices is an ongoing research trend. This paper aims to review issues concerning the driving technologies of WBG semiconductor switching devices. First, a general introduction of the GaN HEMT and the SiC MOSFET is given in the first section. In the second section, a survey of commercial GaN HEMTs above 600 V and SiC MOSFETs between 600 and 1200 V is presented. In the third section, the challenges and solutions of driving GaN HEMTs and SiC MOSFETs are addressed. A survey of commercial drivers designed for WBG switching devices is then provided in the fourth section. The fifth and sixth sections cover the literature review on driving circuits for GaN HEMTs and SiC MOSFETs, respectively. Lastly, this paper is concluded in the seventh section.

## 2. Commercial Wide-Bandgap (WBG) Switching Devices

### 2.1. Discrete Commercial GaN High Electron Mobility Transistors (HEMTs)

According to two famous electronic device providers, Digi-Key [10] and Mouser [11], GaN HEMT products can be purchased from several manufacturers, including EPC (15~200 V) [12], Infineon Technologies (400 and 600 V) [13], GaN Systems (100 and 650 V) [14], Panasonic (600 and 650 V) [15], Nexperia (650 V) [16], and Transphorm (650 and 900 V) [17]. Currently, the two highest voltage ratings of commercial GaN HEMTs are 900 and 650 V, respectively. The 900 V GaN HEMTs are produced by Transphorm, and 650 V GaN HEMTs are produced by GaN Systems, Panasonic, Nexperia (formerly Standard Products business unit of NXP Semiconductors), and Transphorm. Table 3 presents the device specifications of commercial GaN HEMTs above 600 V, where MFR stands for manufacturer, *V_ds_* denotes drain-source voltage, *I_ds_* denotes drain-source current, *V_TH_* denotes threshold voltage, *V_gs_* denotes gate-source voltage, *R_ds(on)_* denotes on resistance, and *C_iss_* denotes input capacitance. Some specifications of the latest large-current devices from GaN Systems are not published. The maximum *V_gs_* values of the products from Infineon Technologies and Panasonic are not specified because these devices are current-controlled, which offers good robustness but leads to higher gate losses.

### 2.2. Discrete Commercial SiC Metal-Oxide-Semiconductor Field-Effect Transistors (MOSFETs)

**Figure 6:**
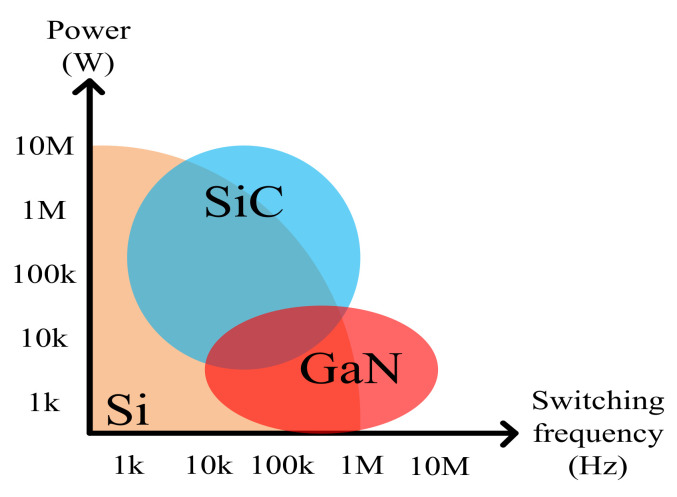
Applications fields of Si, SiC, and GaN switching devices [13].

Since SiC MOSFETs have been developed for a longer time, there is a much larger variety of companies that produce SiC MOSFETs: ON Semiconductor (900 and 1200 V) [18], Littelfuse (600~1700 V) [19], Infineon Technologies (650~1700 V) [13], Cree (650~1700 V) [20], Rohm Semiconductor (650~1700 V) [21], STMicroelectronics (650~1700 V) [22], United Silicon Carbide (650~1700 V) [23], Microchip (700~1700 V) [24], and GeneSiC Semiconductor (1200~3300 V) [25]. Table 4, Table 5, Table 6, Table 7, Table 8, Table 9, Table 10, Table 11 and Table 12 present the device specifications of commercial SiC MOSFETs with voltages ratings between 600 and 1200 V. When comparing the listed commercial devices, we can see that it is common for SiC MOSFETs to possess much higher current capabilities than those of GaN HEMTs, which makes SiC devices more suitable for high-power applications such as high-speed railway, power transmission, industrial drives, smart grid, and wind power generation. On the other hand, GaN devices offer smaller on resistances and input capacitances, which indicates that GaN devices have the potential to yield lower conduction losses and faster switching with such ratings. Therefore, GaN HEMTs are currently applied to improve the efficiencies of mid-voltage and mid-power applications such as switching power supply, solar PV, AC/DC adapter, medical equipment, electric vehicle (EV), and uninterruptible power supply. Figure 6 shows the application fields of Si, SiC, and GaN switching devices [13].

### 2.3. Modular WBG Switching Devices

Some commercial switching modules based on GaN HEMTs can also be found from EPC [12], as numerated in Table 13, where HB stands for half bridge. EPC produces up to 3 kW modules. Consequently, these products are not yet matured for high-power applications.

**Figure 7:**
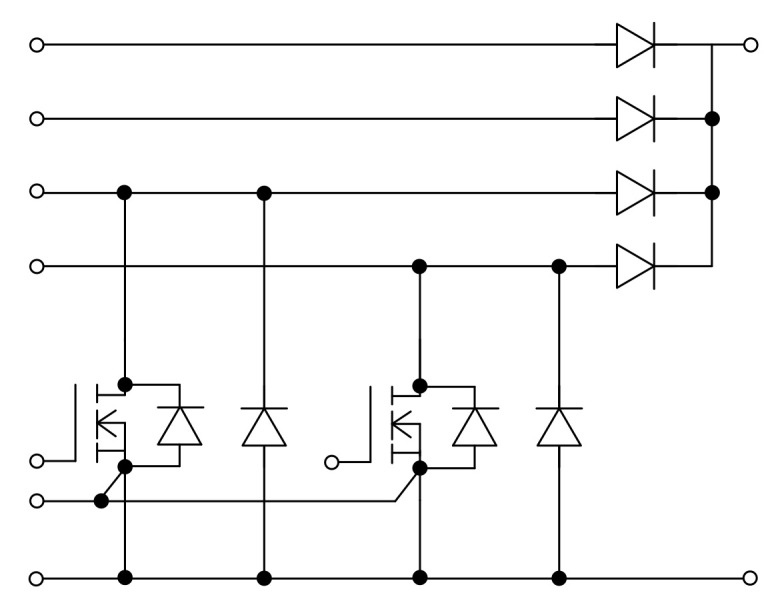
Configurations of Infineon Technologies SiC switching modules DF23MR12W1M1P_B11, DF23MR12W1M1_B11, DF11MR12W1M1P_B11, and DF11MR12W1M1_B11 [13].

Unlike GaN modules, there are several manufacturers with commercial SiC modules available for purchase: Infineon Technologies [13], Cree [20], Rohm Semiconductor [21], Microchip [24], Powerex [26], and SemiQ [27], as listed in Table 14, Table 15, Table 16, Table 17, Table 18 and Table 19, where FB stands for full bridge. As can be imagined, the voltage and current ratings of SiC modules easily exceed those of GaN modules. Among SiC modules manufactured by the abovementioned companies, HB is the most common configuration. Particularly, the configuration of DF23MR12W1M1P_B11, DF23MR12W1M1_B11, DF11MR12W1M1P_B11, and DF11MR12W1M1_B11 by Infineon Technologies is presented in Figure 7. The suitable applications of them are specified as solar applications.

## 3. Considerations for the Design of Driving Circuits for WBG Switching Devices

It has been well accepted that the key factor of realizing WBG switching devices’ full potential is their driving circuits. The main difference in the driving characteristics of WBG and Si switching devices is due to WBG devices’ much faster transient. The fast switching and high switching frequency require shorter driver rise and fall times and propagation delay. Additionally, the slew rate of the WBG devices can reach up to 100 times that of conventional Si devices, which can severely worsen EMI-related problems such as gate ringing and measurement. In order to deal with the fast transient, the driving circuit design and printed circuit board (PCB) layout must be optimized [28,29].

**Figure 8:**
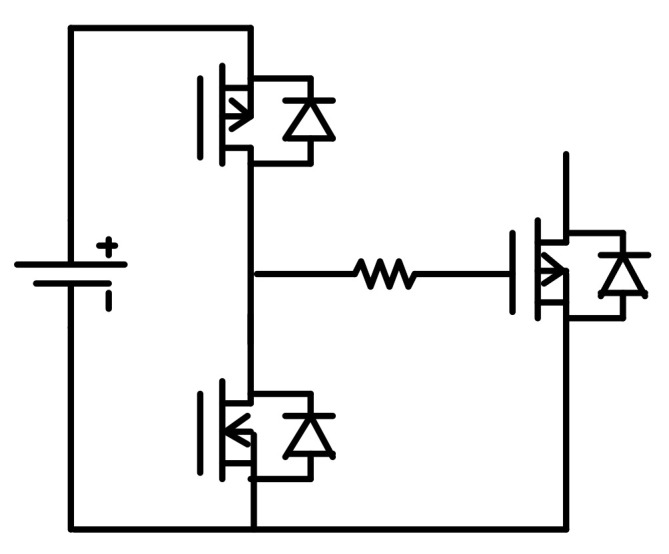
Conventional totem-pole gate driver.

In general, the fundamental rules of driving high-power GaN HEMTs and SiC MOSFETs are to apply high drive strength, provide enough isolation between driving and power circuits, prevent voltage oscillation, limit gate voltage spikes, and optimize dead time. Isolation can be provided by various types of isolators or isolated drivers suitable for WBG switching devices. In general, it is also possible to use high-speed MOSFET and IGBT drivers (similar to Figure 8) to drive WBG devices, but the complexity of the driving circuits and cost may be increased. However, another special characteristic of WBG switching devices is that they do not always use symmetrical driving voltages (such as ±18 and ±20 V), so there is often a need for asymmetric driving voltage design. Next, it is recommended to minimize the parasitic inductance and capacitance by minimizing the length of the driving loop and the overlapping between circuits and using devices with short or no wire bond [30,31,32]. In particular, GaN HEMTs (especially E-mode) have faster transients and more narrow driving voltage ranges, and SiC MOSFETs have higher power ratings. Consequently, the EMI issues are more dominant when driving GaN HEMTs, and higher driving strength is required when driving high-power SiC MOSFETs.

**Figure 9:**
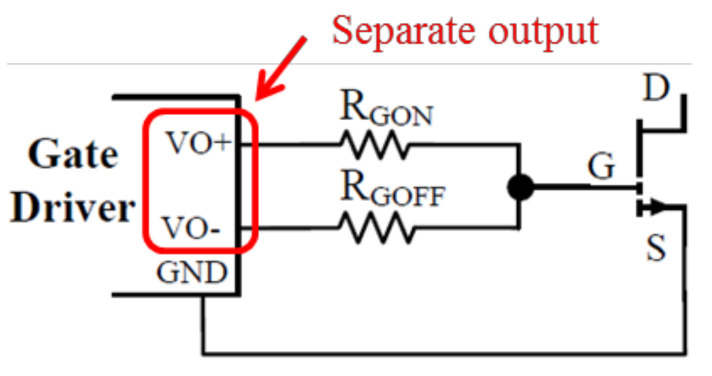
Gate driver with separate high and low outputs [31].

**Figure 10:**
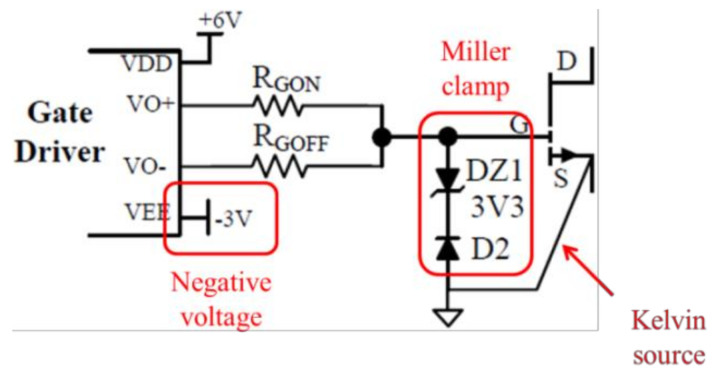
Driving loop with active Miller clamp, Kelvin source connection, and negative turn-off voltage [31].

For the turn-on period, the sum of external resistance and driver’s output resistance should be designed to be much larger than the internal resistance of the power switching device in order to reduce the influence of internal resistance on the switching speed and damp voltage overshoot. If there is a need to damp gate ringing of certain frequencies, ferrite beads can be used as gate impedance as well. However, a low-impedance turn-off path is also required to ensure fast turn-offs and thus prevent shoot-through. As a result, it is usually recommended to design separate turn-on and turn-off paths, where drivers with separate high and low outputs can provide more flexibility, as shown in Figure 9. Moreover, active Miller clamps can be used to directly limit gate-source voltage range; negative turn-off voltage can increase the turn-off speed even more; and Kelvin source connection can separate the driving loop and power loop, so that the influence of parasitic inductance on the driving loop can be minimized, as shown in Figure 10 [31,32,33]. Particularly, active gate drive can be used to control the slew rate directly [34,35].

## 4. Commercial Drivers for WBG Switching Devices

### 4.1. GaN HEMT Drivers

According to Digi-Key [10] and Mouser [11], commercial GaN HEMT drivers are currently available from several companies: Infineon Technologies [13], On Semiconductor [21], Maxim Integrated [36], pSemi [37], Silicon Laboratories [38], and Texas Instruments [39], as given in Table 20, Table 21, Table 22, Table 23, Table 24 and Table 25. It is desirable to use drivers integrated with multiple functions such as digital control and signal detection in order to reduce the number of external devices required. Some companies also produce GaN power modules that integrate GaN HEMTs with designed drivers: EPC [12], Texas Instruments [39], and Navitas Semiconductor [40], as presented in Table 26, Table 27 and Table 28.

### 4.2. SiC MOSFET Drivers

Since SiC devices have been developed for a longer time, a larger variety of SiC MOSFET drivers than that of GaN HEMT drivers have been developed by many manufacturers, including Infineon Technologies [13], On Semiconductor [21], Microchip [24], Maxim Integrated [36], Silicon Laboratories [38], Texas Instruments [39], Analog Devices [41], Tamura [42], Rohm Semiconductor [21], Littelfuse [19], Diodes Incorporated [43], NXP Semiconductors [44], and Power Integrations [45], as listed in Table 29, Table 30, Table 31, Table 32, Table 33, Table 34, Table 35, Table 36 and Table 37. Because SiC MOSFETs are suitable for and often used in high-power applications, the peak output current ratings of SiC MOSFET drivers are generally larger than those of GaN HEMT drivers. Particularly, Tamura’s drivers offer as large as 43 A peak driving current.

## 5. Review on GaN HEMT Driving Circuits

### 5.1. Single-Channel GaN HEMT Driving Circuits

To provide readers with direct design references, typical papers presenting the design of GaN HEMT driving circuits are reviewed in this subsection with examples of single-channel drive. Gurpinar and Castellazzi [46] conducted a benchmark of Si-, SiC-, and GaN-based switching devices at a 600 V class in 3.5 kW, 700 V/230 V, 16~160 kHz single-phase T-type inverter. Evaluated items included gate driver requirements, switching performance, inverter efficiency performance, heat sink volume, output filter volume, and dead-time effect for each technology. A Broadcom gate drive optocoupler ACPL-P346 was selected as the isolated driver for Panasonic PGA26A10DS, and an XP Power isolated DC/DC converter IH0512S-H was used to provide +12 V supply. The design offered small footprint, but the drive strength was limited at 3 A. The series capacitor C_s_ in the proposed driving circuit was designed at 2.82 nF in order to provide −4.5 V during turn-off and speed up turn-on transient. In [47], a low-inductance switching power cell was designed for a three-level ANPC inverter based on GaN System GS66508T. Texas Instruments UCC27511 provided separate turn-on and turn-off outputs. PWM signal was generated using a fiber optic link, and an inverting Schmitt trigger were used to transfer the PWM signal and avoid any false turn-on or turn-off. The signal was then isolated using Silicon Laboratories Si861x. A 7 V power supply was provided using an isolated DC/DC converter and a low-dropout (LDO) regulator. A Schottky diode was used for voltage clamp. The four-layer PCB and surface-mount components significantly reduced the loop inductance using flux cancellation, where the layout was required to eliminate common-mode current circulation. In [48], a 1.5 kW HB bidirectional DC/DC converter was proposed based on GaN System GS66508T driven by Silicon Laboratories Si8271 and −3~6 V driving voltage. A CUI isolated DC/DC converter PES1-S5-S9-M-TR was used to provide power supply. Four-layer PCB layout was adopted in this paper, where flux cancellation was adopted in order to minimize the loop inductance. Advanced Thermal Solutions heat sink ATS-FPX060060013-112-C2-R1 was chosen to match the small thermal pad of GS66508T, and a copper bar was placed between the switching device and the heat sink for enhanced thermal performance.

A 3 kW bidirectional GaN-HEMT DC/DC converter was proposed in [49]. The self-designed driving circuit was designed with an additional NPN bipolar transistor that acted as a voltage clamp and showed no impact on the switching speed. Zero voltage turn-on and negative turn-off voltage were utilized to reach >99% efficiency. A fast GaN HEMT driving circuit was designed for Panasonic PGA26E19BA with a voltage clamped to achieve optimized switching performance, freewheeling conduction, and short-circuit robustness [50]. The manufacturer-recommended driving circuit design was modified by adding a diode-resistor network that helped the capacitor on the driving path quickly discharge as well as provided more flexibility in gate resistor design.

### 5.2. Dual-Channel GaN HEMT Driving Circuits

Dual-channel GaN HEMT driving circuits are generally more complex than single-channel driving circuits. However, there are some commercial dual-channel GaN HEMT drivers ICs that greatly reduce the design complexity. Zhao et al. [51] designed a 2 kVA GaN-based single-phase inverter. The driving circuit for GaN System GS66508T consisted of Analog Devices ADuM7223 and separate turn-on and turn-off paths. The driver IC selection was limited in the writing of this paper (2016), so the selected driver failed to meet the common-mode transient immunity (CMTI) requirement of over 150 V/ns, but no failure occurred during testing. In [52], a 2 kW inductive power transfer system was designed based on a 600 V GaN gate injection transistor (GIT). The slew rate was lowered so that the Analog Devices ADuM3223, which had state of the art CMTI capability of 50 V/ns back in 2016, was compatible. In [53], a high-power density single-phase FB inverter was designed based on GaN Systems GS66502B. Silicon Laboratories Si8274 was used to drive the GaN HEMTs. Ways to minimize parasitic inductance and optimize heat dissipation were discussed, including Kelvin source connection, short and wide PCB trails (preferably copper), minimized overlap between driving loop and power loop, flux cancellation, sufficient area and thickness of copper, small thermal vias but large in number, direct copper and thermal via placement, increased number of layers, and reduced thickness of PCBs.

A totem-pole bridgeless power factor correction (PFC) converter based on GaN Systems GS66508B was proposed in [54]. The Silicon Laboratories Si8273 was used to drive the GaN HEMTs, and Kelvin source connection, voltage clamp, discharging resistor, and separate turn-on and turn-off paths were utilized. Elrajoubi et al. [55] designed an AC/DC converter for battery charging application based on GaN Systems GS66504B. The Silicon Laboratories Si8273 was used to drive the switches, and the Texas Instruments TMS320F28335 was used to generate PWM signals. Kelvin source connection, four-layer PCB, optimized layout, and voltage clamp were taken into consideration.

In [56], a bidirectional buck–boost converter for EV charging application was designed based on 1.3 kV series-stacked GS66508T switching modules. The modules were each designed with two GaN devices, proper gate impedances, and voltage clamps and required only one driving signal to function. The design yielded almost equally shared DC bus voltage.

### 5.3. Brief Summary of Reviewed GaN HEMT Driving Circuits

Table 38 categorizes the reviewed papers according to whether they adopted commercial drivers or self-designed drivers and whether the designed circuits are single-channel or dual-channel circuits, and adopted deriver ICs (if used) are also listed. Sun et al. [57] compared resonant gate drivers for both Si MOSFETs and GaN HEMTs applications. It was concluded that the resonant driver reduced switching losses because of fast charging/discharging capability.

### 5.4. GaN HEMT Power Stages with Integrated Driving Circuits

Switching module design is also a research focus in the field of GaN HEMT driving. A high-power density, high-efficiency half-bridge module based on insulated metal substrate was proposed in [58] for >3 kW applications. In both high side and low side, two GaN System GS66508Bs were paralleled. The 6-layer module consists of four high-side and four low-side switches. The most crucial work was to minimize and balance parasitic inductances of the GaN switching devices. Brothers and Beechner [59] proposed a three-phase 100 V/270 A (per phase) GaN module consisting of six GaN System GS61008Ts. The module layout was then designed based on references from commercial modules and modules in literature. The proposed module successfully hard switched up to 375A.

## 6. Review on SiC MOSFET Driving Circuits

### 6.1. Single-Channel SiC MOSFET Driving Circuits

Since the SiC MOSFET has been developed longer than the GaN HEMT, a lot more papers regarding its driving circuit can be found in open literature. Pirc et al. [60] improved the performance of a nanosecond pulse electroporator by adopting Cree C2M0025120D. RECOM DC/DC converters RP-0512D and RP-0505S were used to provide −5 and 24 V isolated power supply, the Broadcom optocoupler HCPL-0723 was used for signal isolation, and the Littelfuse ultrafast MOSFET driver IXDD609SI was used to drive the SiC MOSFETs. In [61], an isolated smart self-driving multilevel SiC MOSFET driver for fast switching and crosstalk suppression was proposed using variable gate voltage generated with an auxiliary circuit that acted differently during turn-on and turn-off periods. The Isolated Analog Devices single-channel driver ADuM4135 was used to drive Cree C2M0040120D. The designed driving circuit adopted a two-state turn-off scheme. At first, the turn off was ensured using negative voltage, and the voltage was then switched to zero to avoid negative voltage breakdown. The circuit was simple, suitable for integration, highly efficient, compact, and cost-effective. A SiC-based 4MHz 10 kW single-phase zero-voltage-switching inverter for high-density plasma generators was proposed in [62]. The Littelfuse single-channel driver IXRFD630 was used to drive two parallel-connected Cree C2M0080120Ds. The required negative voltage was generated with an auxiliary circuit consisting of a resistor, a capacitor, and a zener diode.

Kim et al. [63] proposed a MHz SiC MOSFET driving circuit using parallel connected FBs based on EPC E-mode device EPC2016. The Broadcom ACPL-346 was used to drive the GaN FBs. The more FBs connected in parallel, the higher the output frequency of pulse width modulation (PWM) could be. The circuit was successfully tested at 2 MHz SiC MOSFET switching frequency using two FBs for a 600 W DC–DC converter. The switching frequency of 5 MHz was also verified achievable using two FBs (2.5 MHz * 2). In [64], a high-speed gate driver was designed focusing on high-temperature capability (180 °C) with low cost, and the circuit was integrated with overcurrent and undervoltage lockout protection. High-temperature transistors (from On Semiconductor), diodes, zener diodes, and pulse transformer were used to realize the design. Even higher operating temperature could be further realized by using polyimide- and hydrocarbon-based PCBs. The cost was successfully reduced from United States dollar (USD) 2250 to 100 compared with using Cissoid EVK-HADES1210. The tested SiC MOSFET was Fuji Electric MT5F31003. Qi et al. [65] developed a 30 kVA three-phase inverter based on Cree SiC HB power module CAS300M12BM2 in order to investigate how to achieve cost-effective outstanding high-temperature performance (targeted at 180 °C ambient temperature). The designed driving circuit was based on Central Semiconductor high-temperature transistors in metal can packages (rated at 200 °C) and consisted of signal isolation, gate drive, saturation detection, undervoltage detection, and protection logic circuits. It achieved 90% cost reduction compared with driving circuits using commercial silicon-on-insulator (SOI) ICs (from USD 2250 to 50 for active components).

A low-cost analog active driver was proposed in [34] for a higher parasitic environment. The designed driver consisted of a current amplifier stage and turn-on and turn-off switching controllers and outputted continuous analog current. Cree C2M0080120D was used to verify that the designed driver reduced losses compared with a hard switched gate driver and that good dynamic was achieved with much larger parasitic inductance and capacitance. In [66], a driving circuit with minimum propagation delay was designed to drive Rohm SCH2080KE for high-temperature applications. The design consisted of signal isolation and level shifting circuits and a two-level isolated auxiliary power supply. The isolation circuit achieved very small propagation delay by using non-delay RC differential circuits and a set-rest flip-flop. The auxiliary power supply was compatible with wide input voltage and operating temperature ranges because of the exclusion of low-temperature linear optocouplers. Li et al. [67] focused on crosstalk elimination. High off-state gate impedance was employed to eliminate the voltage drop on the common-source inductance, while the potential fault turn-on was prevented by utilizing the pre-charged voltage in the gate-source capacitance. Cree C2M0025120D was used to verify the driving circuit, and the design was compatible with most of the commercial SiC MOSFETs. Zhao et al. [68] proposed an intelligent and versatile active gate driver with three turn-on speeds and two turn-off speeds using an adjustable voltage regulator, a voltage selector, and a current sinking circuit. Cree C2M0080120D was used to verify the design.

### 6.2. Dual-Channel SiC MOSFET Driving Circuits

A 1 kW interleaved high-conversion ratio bidirectional DC-DC converter based on four Rohm SiC MOSFET SCTMU001F and four Si MOSFETs was proposed in [69] for distributed energy storage systems. Each driving circuit drove one SiC MOSFET and one Si MOSFET and consisted of Texas Instruments UCC27531, a transformer with two secondary windings, and a voltage clamp. In [70], two series-connected Cree C2M0160120Ds were driven by a low-cost, simple, and reliable driving circuit based on the Broadcom ACPL-344JT, coupling circuits, dv/dt limiting circuit, and a voltage limit circuit. Good voltage balancing and reliable switching were obtained. Lower switching frequency and smaller DC bus voltage were also compatible for the designed circuit. Wang et al. [71] proposed an enhanced gate driver consisting of the STMicroelectronics galvanically isolated MOSFET/IGBT driver STGAP1AS, a bipolar junction transistor-based multi-cell current booster, a high-bandwidth and high-accuracy nonintrusive Rogowski switch-current sensor, and a noise-free isolation architecture. The designed driver was verified with Cree HB module CAS300M17BM2 and compatible with almost all the SiC MOSFET modules. Yang et al. [72] proposed a driving circuit with dynamic voltage balancing for series-connected SiC MOSFETs. Only one external driving IC was required to drive both switches. An overdrive control method helped adapt to DC-bus voltage variation. Switched capacitors could be utilized to further widen the control range. The Cree C2M1000170D was used to verify the design, which was suitable for various high-voltage applications.

A single gate driver was designed to drive four cascaded series-connected SiC MOSFETs for medium voltage applications [73]. This was realized using an auxiliary circuits consisting of diodes, zener diodes, resistors, and capacitors. A 2400 V 10 kHz synchronous boost converter was demonstrated using the designed driving circuit.

### 6.3. Brief Summary of Reviewed SiC MOSFET Driving Circuits

As can be imagined, many similarities related to safety and loss reduction can be observed in GaN HEMT and SiC MOSFET driving circuit designs. A difference is that SiC MOSFETs require higher driving strength and high temperature capability when used in high-power applications. Table 39 categorizes the reviewed papers according to whether they adopted commercial drivers or self-designed drivers and whether the designed circuits are single-channel or multichannel driving circuits, and adopted deriver ICs are also listed. Sakib et al. [74] compared various gate drivers for SiC MOSFETs (2017). Covered aspects included passively triggered gate drive, negative spike mitigation, crosstalk prevention, and resonance and clamping. Another review was conducted on SiC MOSFET devices and individual SiC MOSFET gate drivers (2018) [75]. Covered items included the adjustment of switching speed, voltage, and power level and other special functions. In [76], the status and applications of SiC-based power converters, challenges regarding high-switching frequency gate driver design, and problems related to commercial drivers were reviewed (2018). It was pointed out that the commercial drivers back then were far from universal, which was due to very specific driving requirements of various SiC switching devices. Liu and Yang [77] reviewed the characteristics of SiC MOSFETs and different driving circuits (2019). It was suggested that, for >150 °C applications, discrete components or Si-on-insulator-based gate drivers should be used rather than conventional Si MOSFET drivers. In terms of crosstalk suppressing, it was recommended that a combination of additional capacitors, variable voltage/resistance driver, and auxiliary discharging path could be used. In [78], slew rate control methods for SiC MOSFET active gate drivers were reviewed (2020). Reviewed aspects included the principle of slew rate control, factors that influenced slew rate, and issues induced by high slew rate. Slew rate control methods included variable gate resistance, input capacitance, gate current, and gate voltage. Control strategies included open-loop, measurement-based, estimation-based, and timing-based controls. The advantages and disadvantages of each control strategy were also listed. Next, the conventional and emerging applications of active gate drive were also reviewed, including EMI noise mitigation, dead-time adaption, motor drive, reliability enhancement of SiC MOSFET, and parallel SiC MOSFET connection.

### 6.4. SiC MOSFET Power Stages with Integrated Driving Circuits

Some examples of modular SiC MOSFET power stages can be found in [79,80,81]. Jørgensen et al. [79] proposed a 10 kV single-switch module adopting −5~20 V hard-switched Littlefuse IXRFD630, Kelvin connection, no external gate resistance for the fastest switching speed possible, and low inductance design for better heat dissipation. In [80], a 1200 V/120 A HB module was designed based on a direct bonding copper-stacked hybrid packaging structure for minimized thermal resistance and commutation power loop inductance. The designed module was tested as a 5.5 kW single-phase inverter, yielding 97.7% efficiency, and the power loss was 28.3% less than Cree HB module CAS120M12BM2. A module with adjustable drive strength based on hybrid combination of logics and high temperature capability was proposed in [81]. The Cree bare die SiC MOSFET CPM3-0900-0065B was successfully switched with less than 75 ns rise/fall time from room temperature to over 500 °C. Overshoot and dv/dt were successfully and dynamically controlled.

## 7. Conclusions

The desire of replacing conventional Si-based switching devices with WBG material-based switching devices for higher switching frequency and efficiency has led to intensive research on the driving technologies of GaN HEMT and SiC MOSFET. This paper has addressed the characteristics and operating principles of GaN HEMT and SiC MOSFET. Commercially available products of WBG switching devices with V_ds_ ranging from 600V to 1200 V were explored. GaN HEMTs are currently suitable for low- to mid-power and high-frequency applications because of their ultrafast switching speed and ultralow conduction losses, and SiC MOSFETs are especially suitable for high-power applications because of their high thermal capability. In this paper, the driving requirements of WBG switching devices have been explained, where overcoming high slew rate is the biggest challenge. Commercial drivers designed for WBG switching devices were surveyed. It has been observed that drivers for GaN HEMTs and SiC MOSFETs are normally designed based on their specific system requirements. Finally, typical papers discussing the driving circuits of GaN HEMT and SiC MOSFET, previously published review papers, and some papers focusing on modular design of WBG switching devices integrated with driving circuits have been reviewed with brief discussions.

## Figures and Tables

**Table 1:** General comparison of normally off GaN high electron mobility transistors (HEMTs).

Device	Driving Voltage Threshold	Driving Voltage Range	Operating Temperature	On Resistance	Body Diode
E-mode	<2 V	−10 V~7 V	Higher	Lower	X
Cascode	~4 V	±20 V	Lower	Higher	O

**Table 2:** General comparison of Si metal-oxide-semiconductor field-effect transistors (MOSFET), normally off GaN HEMTs, and SiC MOSFET.

Device	Driving Voltage Strictness	Power Rating	Switching Speed	On Resistance	Operating Temperature	Body Diode
Si MOSFET	4th	2nd	4th	4th	3rd	O
E-GaN	Highest	3rd	Fastest	Lowest	2nd	X
Cascode-GaN	2nd	4th	2nd	2nd	3rd	O
SiC MOSFET	3rd	Highest	3rd	3rd	Highest	O

**Table 3:** Commercial GaN HEMTs above 600 V.

Manufacturer	Type	V_ds_ (V)	I_d__s_ (A)	V_TH_ (V)	V_gs_ (V)	R_ds(on)_ (mΩ)	C_iss_ (pF)
Infineon Technologies	E-mode	600	10	1.2	−10~5	140	157
			12.5				
			15			55	380
			31				
GaN Systems		650	3.5	1.4	−10~7	500	30
			7.5	1.3		200	65
			8	1.4		225	52
			11	1.5		150	74
			15	1.3		100	130
			22			67	195
			30	1.7		50	260
			60	1.3		25	520
			80	-	-	18	-
			150			10	
Panasonic		600	15	3.5	−10~5	140	160
			31			56	405
		650	9.4			270	80
Nexperia	Cascode	650	34.5	3.9	±20	50	1000
			47.2			35	1500
Transphorm		650	6.5	4	±18	240	760
			15		±20	150	576
			16	2.1	±18		720
			20			130	760
			25	4	±20	72	600
			28	2.6	±18		1130
			34	4	±20	50	1000
						60	
			36			50	
			46.5			35	1500
			47				
		900	15	2.1	±18	205	780
			34	3.9	±20	50	980

**Table 4:** Commercial 600~1200 V SiC MOSFETs by On Semiconductor.

V_ds_ (V)	I_d__s_ (A)	V_TH_ (V)	V_gs_ (V)	R_ds(on)_ (mΩ)	C_iss_ (pF)
900	44	2.7	−10~19	60	1800
	46		−10~20		1770
	112	2.6	−10~19	20	4415
	118	2.7			
1200	17	3.1	−15~25	160	665
	17.3				
	19.5	3			678
	29	2.75		80	1112
	30	3			1154
	31	2.7			1112
	58	3		40	1762
	60	3			1789
		2.97			1781
	98	2.7		20	2943
	102				
	103				2890

**Table 5:** Commercial 600~1200 V SiC MOSFETs by Littelfuse.

V_ds_ (V)	I_d__s_ (A)	V_TH_ (V)	V_gs_ (V)	R_ds(on)_ (mΩ)	C_iss_ (pF)
600	15	3	±20	150	2000
1200	22	2.8	−10~25	160	870
	27			120	1125
	39			80	1825
	47	2.6		40	1900
	48	2.8			1895
	68	2.6		25	2790
	90				

**Table 6:** Commercial 600~1200 V SiC MOSFETs by Infineon Technologies.

V_ds_ (V)	I_d__s_ (A)	V_TH_ (V)	V_gs_ (V)	R_ds(on)_ (mΩ)	C_iss_ (pF)
650	20	4.5	−5~23	107	496
	26			72	744
	28				
	39			48	1118
	47			27	2131
	59				
1200	4.7		−7~23	350	182
	13			220	289
	19			140	454
	26			90	707
	36			60	1060
	52		−7~20	45	2130
			−10~20		1900
	56	4.5	−7~23	30	2120

**Table 7:** Commercial600~1200 V SiC MOSFETs by Cree.

V_ds_ (V)	I_d__s_ (A)	V_TH_ (V)	V_gs_ (V)	R_ds(on)_ (mΩ)	C_iss_ (pF)
650	36	2.3	−8~19	60	1020
	37				
	120			15	5011
900	11.5	2.1	−8~18	280	150
	22		−8~19	120	350
	23		−8~18		
	35		−8~19	65	760
					660
	36		−8~18		760
	63	2.4	−8~19	30	1747
1000	22	2.1	−8~19	120	350
	35			65	660
1200	7.2	2.5	−8~19	350	345
	7.6				
	10	2.6	−10~25	280	259
	17	2.8	−8~19	160	632
	18	2.9	−10~25		606
	30	2.5	−8~19	75	1350
					1390
	36	2.9	−10~25	80	1130
	60	2.6		40	1893
	63	2.5	−8~19	32	3357
	90	2.6	−10~25	25	2788
	100	2.5	−8~19	21	4818
	115			16	6085

**Table 8:** Commercial 600~1200 V SiC MOSFETs by Rohm Semiconductor.

V_ds_ (V)	I_d__s_ (A)	V_TH_ (V)	V_gs_ (V)	R_ds(on)_ (mΩ)	C_iss_ (pF)
650	21	2.7	−4~26	120	460
	29	2.8	−10~26	120	1200
	30	2.7	−4~26	80	571
	39			60	852
	70			30	1526
	93			22	2208
	118			17	2884

**Table 9:** Commercial 600~1200 V SiC MOSFETs by STMicroelectronics.

V_ds_ (V)	I_d__s_ (A)	V_TH_ (V)	V_gs_ (V)	R_ds(on)_ (mΩ)	C_iss_ (pF)
650	45	3.2	−10~22	45	1370
				55	
				75	
	90			18	3300
	95	3.1		20	3315
	100				
	116	3.2		15	3380
	119			18	
1200	12	3.5	−10~25	520	290
					300
	20			189	650
	33	3.2	−10~22	75	1230
	45	3.5	−10~25	90	1700
	52	3.1	−10~22	45	2086
	65	3	−10~25	59	1900
	75	3.1	−10~22	30	3400
	91	3.45		21	3540

**Table 10:** Commercial 600~1200 V SiC MOSFETs by United Silicon Carbide.

V_ds_ (V)	I_d__s_ (A)	V_TH_ (V)	V_gs_ (V)	R_ds(on)_ (mΩ)	C_iss_ (pF)
650	18	5	±25	34	1500
				45	
	25			80	
				111	
	31			80	
	41			42	
	54				
	65			27	
	85				
	120	4.7	±20	6.7	8360
1200	7.6	4.7	±25	410	740
	18.4	4.4		150	738
	33	5		80	1500
	34.5			70	
	65			35	
	107	4.7	±20	16	7824
	120			8.6	8512

**Table 11:** Commercial 600~1200 V SiC MOSFETs by Microchip.

V_ds_ (V)	I_d__s_ (A)	V_TH_ (V)	V_gs_ (V)	R_ds(on)_ (mΩ)	C_iss_ (pF)
700	25	2.4	−10~23	90	785
	28			86	
	37			60	1175
	39				
	65	2.7		35	2010
	77				
	126	2.4		15	4500
	140		−10~25		
1200	35	2.8	−10~23	80	838
	37				
	53		−10~25	40	1990
	64	2.6	−10~23	40	1990
	66	2.7			
	77	2.8	−10~25	25	3020
	89		−10~23		
	103				

**Table 12:** Commercial 600~1200 V SiC MOSFETs by GeneSiC.

V_ds_ (V)	I_d__s_ (A)	V_TH_ (V)	V_gs_ (V)	R_ds(on)_ (mΩ)	C_iss_ (pF)
1200	8	3	−10~25	350	225
	16			160	493
	32			75	1053
	33				
	57			40	1974
	59				
	74			30	2633
	78				
	95			20	3949
	107				

**Table 13:** Commercial GaN HEMT modules by EPC.

Configuration	Voltage Rating (V)	Current Rating (A)
HB	30	10/40
		16
	60	10/40
		30
	80	10/40
		30
	100	1.7
		30
HB + bootstrap	60	1.7/0.5
	100	
Dual common source	120	3.4

**Table 14:** Commercial SiC MOSFET modules by Infineon Technologies.

Configuration	Voltage Rating (V)	Current Rating (A)
HB	1200	25
		50
		100
		150
		200
		250
		375
		500
FB		50
HB (3-arm)		25
Figure 7		25
		50
Vienna rectifier phase leg		75
		100

**Table 15:** Commercial SiC MOSFET modules by Cree.

Configuration	Voltage Rating (V)	Current Rating (A)
HB	1200	20
		50
		120
		225
		300
		325
		400
		425
		450
Three-phase		20
		50

**Table 16:** Commercial SiC MOSFET modules by Rohm Semiconductor.

Configuration	Voltage Rating (V)	Current Rating (A)
HB	1200	80
		134
		180
		204
		250
		300
		358
		397
		576
Chopper		134
		180
		204
		300
		358
		576

**Table 17:** Commercial SiC MOSFET modules by Microchip.

Configuration	Voltage Rating (V)	Current Rating (A)
HB	700	124
		241
		353
	1200	55
		89
		173
		254
		337
		495
		733
		805
		947
	1700	50
		100
		280
FB	700	98
	1200	55
		89
		173
HB (3-arm)	700	98
		189
		278
	1200	89
		171
		251
Chopper	700	98
	1200	55
		89
		173
		254
Vienna rectifier phase leg	700	124
		238

**Table 18:** Commercial SiC MOSFET modules by Powerex.

Configuration	Voltage Rating (V)	Current Rating (A)
Split dual SiC MOSFET	1200	100
Dual MOSFET	1700	540

**Table 19:** Commercial SiC MOSFET modules by SemiQ.

Configuration	Voltage Rating (V)	Current Rating (A)
HB	1200	160
		200
		240
		320
HB (2-arm)		40
		80
FB		20
HB (3-arm)		20

**Table 20:** Commercial GaN HEMT drivers by Infineon Technologies.

Num. of Channels	Peak Source Current (A)	Peak Sink Current (A)	Supply Voltage (V)	Rise Time (ns)	Fall Time (ns)	Prop. Delay (ns)
1	4	8	3~20	6.5	4.5	41
2	1	2				37
	4	8				

**Table 21:** Commercial GaN HEMT driver by On Semiconductor.

Num. of Channels	Peak Source Current (A)	Peak Sink Current (A)	Supply Voltage (V)	Rise Time (ns)	Fall Time (ns)	Prop. Delay (ns)
2	1	1.3	9~17	1	1	25

**Table 22:** Commercial GaN HEMT drivers by Maxim Integrated.

Num. of Channels	Peak Source Current (A)	Peak Sink Current (A)	Supply Voltage (V)	Rise Time (ns)	Fall Time (ns)	Prop. Delay (ns)
1	3	7	4~14	4~37	4~18	8
	4	2.85	3~36	3.6	2.5	35
		5.7	−16~36		1.8	53

**Table 23:** Commercial GaN HEMT drivers by pSemi.

Num. of Channels	Peak Source Current (A)	Peak Sink Current (A)	Supply Voltage (V)	Rise Time (ns)	Fall Time (ns)	Prop. Delay (ns)
2	2	4	4~6.5	1	1	11
			4~6	0.9	0.9	9.1

**Table 24:** Commercial GaN HEMT drivers by Silicon Laboratories.

Num. of Channels	Peak Source Current (A)	Peak Sink Current (A)	Supply Voltage (V)	Rise Time (ns)	Fall Time (ns)	Prop. Delay (ns)
1	0.3	0.5	6.5~24	20	20	60
	1.5	2.5				
	2.8	3.4	2.8~30	5.5	8.5	40
2	0.25	0.5	3~30	20	20	30
	0.4	0.6	6.5~30	5.5	8.5	40
	1.8	4	2.5~30	10.5	13.3	45
	2		2.5~24	12	12	30
			3~30			

**Table 25:** Commercial GaN HEMT drivers by Texas Instruments.

Num. of Channels	Peak Source Current (A)	Peak Sink Current (A)	Supply Voltage (V)	Rise Time (ns)	Fall Time (ns)	Prop. Delay (ns)
1	1.3	7.6	4~12.6	12	3	12
	4	4	4.5~18	9	7	13
				8	7	17
		6	4~18	9	4	14
			3~18	5	6	27
		8	4.5~18	9	7	13
	5	5	3~33	10	10	65
	7		4.75~5.25	0.4	0.4	2.5
				0.65	0.85	2.9
2	1.2	5	4.5~5.5	7	3.5	30
						35
	1.5	2.5	3~18	8	9	28
		3	3.8~18	0.5	0.5	10
	4	6	3~18	5	6	25
						28
			3~25	6	8	19
					7	
	5	5	4.5~18	7	6	13
				9	6	17

**Table 26:** Commercial switch-driver-integrated module by EPC.

Configuration	Voltage Rating (V)	Current Rating (A)	Supply Voltage (V)
HB	70	12.5	11~13

**Table 27:** Commercial switch-driver-integrated module by Texas Instruments.

Configuration	Voltage Rating (V)	Current Rating (A)	Supply Voltage (V)
Single switch	600	17	9.5~18
		40	9.5~18
		34	9.5~18
HB	80	10	4.57~5.25

**Table 28:** Commercial switch-driver-integrated module by Navitas Semiconductor.

Configuration	Voltage Rating (V)	Current Rating (A)	Supply Voltage (V)
Single switch	650	5	5.5~24
		8	
		12	

**Table 29:** Commercial SiC MOSFET drivers by Infineon Technologies.

Num. of Channels	Peak Source Current (A)	Peak Sink Current (A)	Supply Voltage (V)	Rise Time (ns)	Fall Time (ns)	Prop. Delay (ns)
1	2	2	−12~28	34	50	170
	4	3.5	3.1~35	10	9	125
	4.4	4.1	3.1~18	9	6	300
	10	9.4	3.1~35	10	9	125
2	2	2	−12~28	30	50	170
	4	8	3~20	6.5	4.5	37

**Table 30:** Commercial SiC MOSFET drivers by On Semiconductor.

Num. of Channels	Peak Source Current (A)	Peak Sink Current (A)	Supply Voltage (V)	Rise Time (ns)	Fall Time (ns)	Prop. Delay (ns)
1	6	6	−8~22	8	8	25
	7.8	7.1	−10~24	10	15	66
2	1.9	2.3	0~20	13	8	90

**Table 31:** Commercial SiC MOSFET drivers by Microchip.

Num. of Channels	Peak Source Current (A)	Peak Sink Current (A)	Supply Voltage (V)	Rise Time (ns)	Fall Time (ns)	Prop. Delay (ns)
2	10	10	14~16	80	90	250
	20	20				

**Table 32:** Commercial SiC MOSFET drivers by Maxim Integrated.

Num. of Channels	Peak Source Current (A)	Peak Sink Current (A)	Supply Voltage (V)	Rise Time (ns)	Fall Time (ns)	Prop. Delay (ns)
1	4	2.85	3~36	3.6	2.5	35
		5.7	−16~36		1.8	53

**Table 33:** Commercial SiC MOSFET drivers by Silicon Laboratories.

Num. of Channels	Peak Source Current (A)	Peak Sink Current (A)	Supply Voltage (V)	Rise Time (ns)	Fall Time (ns)	Prop. Delay (ns)
1	2.8	3.4	0~30	5.5	8.5	40
	4	4	3~30	12	12	19
2	1.8	4	2.5~30	10.5	13.3	45
	4		3~30	12	12	19
						89
						39

**Table 34:** Commercial SiC MOSFET drivers by Texas Instruments.

Num. of Channels	Peak Source Current (A)	Peak Sink Current (A)	Supply Voltage (V)	Rise Time (ns)	Fall Time (ns)	Prop. Delay (ns)
1	1.5	2	−13~33	28	25	70
	2.5	5	−15~30	18	20	76
			0~30			
			−5~32	15	7	17
	4.5	5.3	−13~33	28	25	70
	8.5	10	−16~33	10	10	65
	10		−5~15	33	27	90
			−5~15	28	24	90
			−16~33	10	10	65
			3~33			
	15	15	−12~30	150	150	150
	17	17	−16~33	10	10	65
2	4	6	3~25	6	7	19

**Table 35:** Commercial SiC MOSFET drivers by Analog Devices.

Num. of Channels	Peak Source Current (A)	Peak Sink Current (A)	Supply Voltage (V)	Rise Time (ns)	Fall Time (ns)	Prop. Delay (ns)
1	0.2	0.2	4.5~17	15	15	60
	2	2	2.5~35	18	18	38
			3.3~35	17	17	30
	2.3	2.3	2.5~35	18	18	43
	4	4	3~18	12	12	46
				22	22	53
			−15~30	16	16	55
			−15~35			
	6	6	4.5~25	-	-	107
			6~25	-	-	100
2	0.1	0.1	4.5~18	25	25	124
		0.3	4.5~18.5		10	100
	4	4	3~18	12	12	47
						46
			4.5~18	14	14	160

**Table 36:** Commercial SiC MOSFET drivers by Tamura.

Num. of Channels	Peak Source Current (A)	Peak Sink Current (A)	Supply Voltage (V)	Rise Time (ns)	Fall Time (ns)	Prop. Delay (ns)
2	1.8	1.8	13.5~26.4	-	-	90
	2.5	3.5		-	-	
	3	3				
	4	4		-	-	
	4.5	4.5		-	-	
	6	6		-	-	
	7	7		-	-	
	18	18		-	-	80
	43	43		-	-	100

**Table 37:** Commercial SiC MOSFET drivers by Rohm Semiconductor, Littelfuse, Diodes Incorporated, NXP Semiconductors, and Power Integrations.

MFR	Num. of Channels	Peak Source Current (A)	Peak Sink Current (A)	Supply Voltage (V)	Rise Time (ns)	Fall Time (ns)	Prop. Delay (ns)
Rohm	1	>4 (self-limited)	>4 (self-limited)	4.5~20	15	15	65
Littelfuse		9	9	−10~25	10	10	75
Diodes		10	10	40	48	35	10
NXP		15	15	−12~40	-	-	-
Power Int.		8	8	4.75~28	113	105	270

**Table 38:** Reviewed GaN HEMT driving circuits.

Ref	Channel per Driver	*f_sw_* (kHz)	System *P_out_*	System Efficiency	GaN HEMT	Commercial Driver
[46]	1	160	2.5 kW	97.3%	PGA26A10DS	ACPL-P346
[47]		10	1 kW	unrevealed	GS66508T	UCC27511
[48]		100	1.5 kW	>97%	GS66508T	Si8271
[49]		120~200	3 kW	>99%	GS66508T	N/A
[50]		200	N/A	N/A	PGA26E19BA	
[51]	2	100	2 kVA	97.4%	GS66508T	ADuM7223
[52]		100~250	2 kW	95%	unrevealed	ADuM3223
[53]		160	500 W	96.2%	GS66502B	Si8274
[54]		-	800 W	98%	GS66508B	Si8273
[55]		65~100	1.5 kW	90%	GS66504B	Si8273
[56]		25	unrevealed	unrevealed	GS66508T	N/A

**Table 39:** Reviewed SiC MOSFET driving circuits.

Ref	Channel per Driver	*f_sw_*	System *P_out_*	System Efficiency	SiC MOSFET	Commercial Driver
[60]	1	N/A	unrevealed	N/A	C2M0025120D	IXDD609SI
[61]		1 MHz	51 kW	unrevealed	C2M0040120D	ADuM4135
[62]		4 MHz	10 kW	>97.5%	C2M0080120D	IXRFD630
[34]		20 kHz	5.9 kW	unrevealed	C2M0080120D	N/A
[63]		2 MHz	600 W	unrevealed	C2M0080120D	
[64]		150 kHz	4 kW	>99%	MT5F31003	
[65]		10 kHz	30 kW	99%	CAS300M12BM2	
[66]		100 kHz	2 kW	82%	SCH2080KE	
[67]		unrevealed	4.5 kW	unrevealed	C2M0025120D	
[68]		unrevealed	9 kW	unrevealed	SCH2080KE	
[69]	2	200 kHz	1 kW	96%	SCTMU001F	UCC27531
[70]		25 kHz	2.5 kW	N/A	C2M0160120D	ACPL-344JT
[71]		100 kHz	252 kW	99.4%	CAS300M17BM2	STGAP1AS
[72]		100 kHz	1.8 kW	unrevealed	C2M1000170D	unrevealed
[73]		10 kHz	unrevealed	unrevealed	unrevealed	N/A

## Data Availability

No new data were created or analyzed in this study. Data sharing is not applicable to this article.
